# Megalin and Vitamin D Metabolism—Implications in Non-Renal Tissues and Kidney Disease

**DOI:** 10.3390/nu14183690

**Published:** 2022-09-07

**Authors:** Sono S. Khan, Martin Petkovich, Rachel M. Holden, Michael A. Adams

**Affiliations:** 1 Department of Biomedical and Molecular Sciences, Queen’s University, Kingston, ON K7L 3V6, Canada; 2 Department of Medicine, Queen’s University, Kingston, ON K7L 3V6, Canada

**Keywords:** megalin, 25(OH)D_3_, 1,25(OH)_2_D_3_, vitamin D, chronic kidney disease, parathyroid gland

## Abstract

Megalin is an endocytic receptor abundantly expressed in proximal tubular epithelial cells and other calciotropic extrarenal cells expressing vitamin D metabolizing enzymes, such as bone and parathyroid cells. The receptor functions in the uptake of the vitamin D-binding protein (DBP) complexed to 25 hydroxyvitamin D_3_ (25(OH)D_3_), facilitating the intracellular conversion of precursor 25(OH)D_3_ to the active 1,25 dihydroxyvitamin D3 (1,25(OH)_2_D_3_). The significance of renal megalin-mediated reabsorption of 25(OH)D_3_ and 1,25(OH)_2_D_3_ has been well established experimentally, and other studies have demonstrated relevant roles of extrarenal megalin in regulating vitamin D homeostasis in mammary cells, fat, muscle, bone, and mesenchymal stem cells. Parathyroid gland megalin may regulate calcium signaling, suggesting intriguing possibilities for megalin-mediated cross-talk between calcium and vitamin D regulation in the parathyroid; however, parathyroid megalin functionality has not been assessed in the context of vitamin D. Within various models of chronic kidney disease (CKD), megalin expression appears to be downregulated; however, contradictory results have been observed between human and rodent models. This review aims to provide an overview of the current knowledge of megalin function in the context of vitamin D metabolism, with an emphasis on extrarenal megalin, an area that clearly requires further investigation.

## 1. Introduction

**Figure 1:**
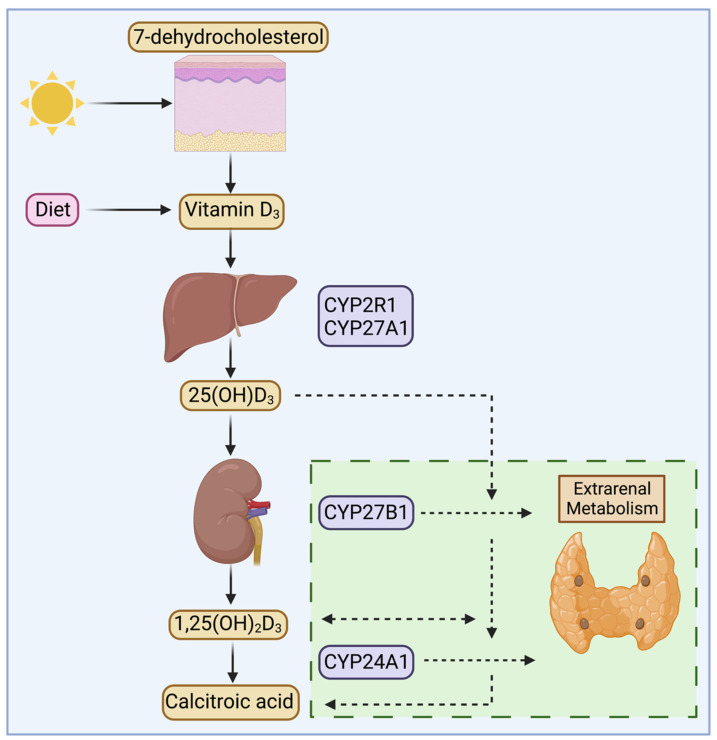
General overview of vitamin D metabolism. Vitamin D_3_ can be obtained after ultraviolet-mediated conversion of 7-dehydrocholesterol or from dietary sources. It then undergoes hydroxylation at the 25-position primarily through the enzymes cytochrome P450 (CYP) 2R1 or CYP27A1 to generate 25 hydroxyvitamin D_3_ (25(OH)D_3_). Maintenance of circulating levels of 1,25 dihydroxyvitamin D_3_ (1,25(OH)_2_D_3_) is primarily achieved after CYP27B1-mediated 1α-hydroxylation of 25(OH)D_3_ in the kidney, and CYP24A1 promotes catabolism of vitamin D compounds. Recent evidence shows that CYP27B1 and CYP24A1 are expressed in extrarenal calciotropic tissues, such as the parathyroid gland, raising possibilities for local, tissue-specific vitamin D activation, action, and inactivation.

Vitamin D is a steroid hormone that plays several critical roles in the body, including the regulation of systemic calcium and bone metabolism. It can be produced by the skin or ingested from the diet, after which it undergoes two consecutive hydroxylation steps. Firstly, in the liver, to 25(OH)D_3_ by mitochondrial or microsomal 25-hydroxylases cytochrome P450 (CYP) 2R1 or CYP27A1. Secondly, 25(OH)D_3_ is transported to the kidney, where it is hydroxylated to 1,25(OH)_2_D_3_ via the 1α-hydroxylase CYP27B1 [1]. The levels of 25(OH)D_3_ and 1,25(OH)_2_D_3_ in circulation appear to be primarily regulated by the 24-hydroxylase CYP24A1, which catabolizes them to 24,25 dihydroxyvitamin D_3_ (24,25(OH)_2_D_3_) or 1,24,25 trihydroxyvitamin D_3_ (1,24,25(OH)_3_D_3_) allowing for further catabolism into calcitroic acid and subsequent excretion in the urine [2,3] (Figure 1). Circulating vitamin D_3_ metabolites are transported by the 58 kDa vitamin D Binding Protein (DBP). DBP possesses the greatest affinity for 25(OH)D_3_, followed by 24,25(OH)_2_D_3_, 1,25(OH)_2_D_3_, and unhydroxylated vitamin D_3_ in order of greatest to least affinity [4,5,6]. As a result of DBP’s high circulating concentration and affinity for 25(OH)D_3_, virtually all circulating 25(OH)D_3_ molecules are DBP-bound [6]. Although the affinity of DBP for 1,25(OH)_2_D_3_ is lower than 25(OH)D_3_, the exceptionally high concentration of DBP in circulation also suggests that most circulating 1,25(OH)_2_D_3_ is DBP-bound. As such, the free concentrations of 25(OH)D_3_ and 1,25(OH)_2_D_3_ are 10 pM and 1 pM, respectively, representing <0.1% and ~1% of their total circulating concentrations [7,8]. 

Megalin, an endocytic receptor abundantly expressed in the proximal tubular epithelial cells (PTEC) of the kidney, functions in the uptake of DBP complexed to 25(OH)D_3_. This uptake facilitates the intracellular conversion of the 25(OH)D_3_ precursor to the active form, 1,25(OH)_2_D_3_. This role of megalin in the reabsorption of DBP-bound vitamin D metabolites from the renal proximal tubule in the kidney has been well established. 1,25(OH)_2_D_3_ or calcitriol, is the most biologically active form of vitamin D; however, other vitamin D metabolites have been implicated in conferring various biological functions [9]. 

The kidney was initially thought to be the sole organ responsible for producing active 1,25(OH)_2_D_3_, but it is now recognized that the expression of CYP27B1 is widespread, including in calciotropic tissues, such as the parathyroid gland [10]. Thus, although the kidney is the primary source for 1,25(OH)_2_D_3_ in the circulation, the presence of extrarenal CYP27B1 underscores the potential for local, tissue-specific 1,25(OH)_2_D_3_ production [11,12]. The actions of vitamin D can be broadly divided into two categories: (1) those that regulate calcium and phosphate homeostasis, termed the ‘classical’ actions, and (2) the ‘non-classical’ actions, which can affect inflammation, immune function, anti-oxidation, and anti-fibrosis, among many others [13,14,15,16]. Given its importance in nutrition, Vitamin D homeostasis has been studied in many diseases where low levels of serum DBP, 25(OH)D_3_, or 1,25(OH)_2_D_3_ have been implicated in various conditions including cancers, cardiovascular diseases, diabetes, inflammatory and autoimmune diseases [17,18,19,20]. Vitamin D primarily exerts its actions through the nuclear vitamin D receptor (VDR), which is expressed in nearly all tissues, with genomic screens identifying thousands of vitamin D response elements (VDREs) throughout the genome controlling hundreds of genes [21]. CYP27B1 expression has been identified in numerous epithelia, the placenta, various bone cells, various cells of the immune system, and endocrine glands, such as the parathyroid and thyroid glands [22,23,24,25,26]. A complete list has been summarized by Bikle et al.; however, the physiological relevance of extrarenal tissues expressing CP27B1 has not been fully elucidated, and it is unclear if 1,25(OH)_2_D_3_ influences hormone release in the thyroid gland [12]. Megalin is also expressed in all of these tissues, suggesting an interconnected role between megalin-mediated uptake of vitamin D and the action of vitamin D-metabolizing enzymes in extrarenal tissues, such as the parathyroid gland [27]. The role of megalin in the metabolism of vitamin D in the kidney and extra-renal tissues is the focus of this review.

## 2. Megalin and Cubilin

### 2.1. Megalin Overview

**Figure 2:**
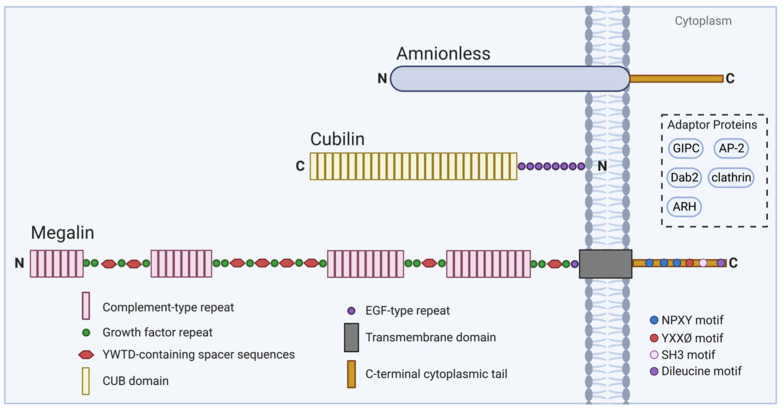
Schematic of megalin and cubilin structure and associated molecules. The structure of megalin plays an essential role in its function, performing receptor-mediated endocytosis. The extracellular domain contains complement-type repeats with ligand-binding properties, YWTD-containing spacer sequences which function in the pH-dependent release of ligands, and other cooperating repeat sequences. The C-terminal cytoplasmic tail contains several motifs, such as NPXY, which are essential for proper megalin trafficking. Megalin trafficking may be enhanced through the interaction of intracellular adaptor proteins like GIPC, AP-2, Dab2, clathrin, and ARH with motifs in the cytoplasmic tail of megalin. Megalin function may also be enhanced through cooperative interaction with cubilin and amnionless (AMN).

Megalin is a large transmembrane glycoprotein of approximately 600 kDa (4665 amino acids) with homology to the human low-density lipoprotein receptor family [28,29,30]. The extracellular domain of human megalin has ligand-binding properties, due to the following three kinds of repeats: (1) four clusters of cysteine-rich complement-type repeats, representing the ligand-binding domains; (2) 16 growth factor repeats separated by eight spacer sequences containing the tetrapeptide YWTD (Tyr-Trp-Thr-Asp), functioning in the pH-dependent release of ligands; and (3) one epidermal growth factor (EGF) repeat [30]. Megalin possesses a single transmembrane domain of 23 amino acids and a C-terminal cytoplasmic tail of 209 amino acids [31]. Of interest are three NPXY (Asn-Pro-X-Tyr) motifs found in the cytoplasmic tail. Upon deletion of the second NPXY-like motif, megalin trafficking is impaired, and deletion of the first and third NPXY motifs diminishes effective megalin-mediated endocytosis [31]. Binding of the NPXY motifs to cytoplasmic adaptor proteins, such as ARH, AP-2, clathrin, Dab2, and GIPC, may promote the proper assembly of endocytic compartments, megalin trafficking and ligand-selectivity, and signal transduction, among other essential functional roles [32,33,34,35,36] (Figure 2). 

In the kidney, megalin is abundantly expressed in the brush border of apical membranes of PTECs, endocytic vessels, microvilli, dense apical tubules, glomerular podocytes, and within lysosomes to a lesser extent [37,38,39,40,41]. However, megalin is also widely distributed and has been identified in several extrarenal absorptive epithelia, including the choroid plexus, the visceral yolk sac, the ciliary body and retinal pigment epithelium of the eye, gall bladder, placenta, ependymal cells, the epididymis, type II pneumocytes within the lung alveoli, the epithelium of the small intestine, and within the thyroid and parathyroid glands [38,42,43,44,45,46]. The widespread expression of megalin is consistent with its purported role as a multiligand scavenger receptor, as several physiologically relevant substrates have been identified as ligands for megalin, including albumin, hemoglobin, insulin, retinol-binding protein (RBP), and, of direct importance to this review, vitamin D-binding protein (DBP) [47,48,49,50,51]. A comprehensive list of megalin ligands has been summarized by Nielsen et al. [27]. This review focuses on the role of extrarenal megalin in the context of vitamin D metabolism, and how parathyroid megalin may have direct implications for disease.

### 2.2. Cubilin and Associated Molecules in Endocytosis

Cubilin, although not the primary focus of this review given the limited literature regarding its role in vitamin D metabolism, is a 460-kDa proximal tubular endocytic receptor that binds DBP and cooperates closely, and colocalizes with megalin [52]. Cubilin and megalin are co-expressed in apical epithelial cells and colocalize in the endocytic apparatus of absorptive epithelia in the intestine, kidney, yolk sac and gallbladder, among other tissues [44,46,53,54]. The structure of cubilin suggests an essential functional relationship to megalin, as cubilin has neither a transmembrane domain nor a cytoplasmic tail but is anchored to the membrane via a complex with the membrane protein amnionless (AMN) and megalin [55,56,57]. The binding of cubilin to megalin has been shown by in-vitro and in-vivo studies to be Ca^2+^-dependent, with binding significantly reduced in the presence of EDTA, a Ca^2+^-chelating agent. Functional cubilin was immunoprecipitated in complex with AMN and megalin from renal brush border membranes, and silencing of either megalin or AMN showed an 85–90% reduction in cubilin expression and a 2-fold decrease in its half-life, suggesting that the interaction of cubilin with both megalin and AMN is essential for its intracellular stability [57]. 

### 2.3. Megalin and Cubilin Interactions

Megalin-knockout mice demonstrate decreased cubilin expression and ligand uptake, and antibodies against megalin inhibit cubilin membrane association and increase degradation by 50–60%, reflecting a meaningful functional relationship between megalin and cubilin [58,59]. However, as indicated by a case study on a patient with a cubilin deficiency, cubilin dysfunction does not impair megalin-mediated endocytosis but exacerbates the loss of shared ligands, such as DBP [60]. Interestingly, thyroidectomized rats showed a 70% reduction in kidney levels of cubilin, and mucosal membrane cubilin associated with megalin was reduced by ~66% relative to controls, independent of megalin levels. These effects were reversed upon thyroxine treatment, where cubilin associated with megalin increased by 100% relative to controls, suggesting a thyroxine-mediated mechanism of regulation of megalin and cubilin association [45]. Cubilin has reduced function in the absence of megalin, as indicated by a kidney-specific knockout, where the cubilin-AMN complex promoted endocytosis of intrinsic factor-vitamin B12 and albumin, but megalin considerably increased this uptake by driving internalization of cubilin-ligand complexes [46,61]. As such, megalin and cubilin form a coreceptor complex in which cubilin likely sequesters specific ligands on the cell surface, enabling megalin-mediated internalization of the cubilin-bound ligand complex [62]. This has immediate relevance to vitamin D metabolism, as megalin, similarly to cubilin, demonstrates Ca^2+^-dependent binding to DBP, with K_d_ values of 110 ± 15 nM and 120 ± 27 nM for cubilin, and megalin, respectively [62]. As such, cubilin in dogs with impaired cubilin biosynthesis did not effectively colocalize with megalin and had reduced, but not abolished, endocytosis of DBP [62]. Conversely, megalin knockout mice showed no DBP endocytosis despite intact expression of cubilin. Nevertheless, circulating vitamin D metabolites (25(OH)D_3_ and 1,25(OH)_2_D_3_) were reduced by approximately 50% in the cubilin-deficient dogs, highlighting a potentially important role of cubilin in vitamin D homeostasis [62]. Similar disruptions in DBP and circulating vitamin D handling were observed in human patients with cubilin mutations [62,63]. 

## 3. Megalin and Vitamin D Metabolism in the Kidney

In the kidney, 25(OH)D_3_ conversion to 1,25(OH)_2_D_3_ occurs primarily within the mitochondria of PTECs [64,65]. Megalin is abundantly expressed in the brush border of apical membranes of PTECs, endocytic vessels, microvilli, dense apical tubules, glomerular podocytes, and within lysosomes to a lesser extent [37,38,39,40,41]. For PTECs to sense and respond to systemic changes and demands for 1,25(OH)_2_D_3_ production, the precursor 25(OH)D_3_ must gain intracellular access. One theory, the free hormone hypothesis, states that the biological activity of vitamin D metabolites, including 25(OH)D_3_ and 1,25(OH)_2_D_3_, are mediated by their unbound (DBP-free) forms in circulation, which enter target cells through passive diffusion and not via an active transport mechanism [66]. Support for the free hormone hypothesis comes from a DBP knockout mouse model in which calcium homeostasis was not significantly altered despite there being severe circulating 1,25(OH)_2_D_3_ deficiency [67]. However, CYP27B1 and CYP24A1 activities were significantly upregulated and downregulated, respectively, in the kidneys of DBP-null mice, suggesting a compensatory mechanism for the lack of DBP to maintain 1,25(OH)_2_D_3_ levels [67]. 

While the free hormone hypothesis purports vitamin D metabolites enter PCT cells through passive diffusion, this was challenged by the findings of Nykjaer et al. (1999), who demonstrated that the 25(OH)D_3_-DBP complex is filtered through the glomerulus and reabsorbed in PTECs by megalin [51]. Endocytosis was required to preserve the systemic DBP concentration, as urinary DBP was exclusively observed in megalin-knockout animals but not controls [51]. The absence of renal megalin abolished any binding or uptake of endogenous DBP in the kidney, underscoring megalin as the primary renal DBP receptor [51]. Exposure to receptor-associated protein (RAP), a specific inhibitor of megalin-mediated endocytosis [68,69], decreased DBP uptake into the kidney to 8.2% of untreated controls, with the remainder excreted into the urine, suggesting that megalin has a functionally relevant role in the uptake of DBP and vitamin D-DBP complexes [51]. Further, endocytosis was required to preserve the systemic 25(OH)D_3_ concentration, as levels of plasma 25(OH)D_3_ were reduced by 80% and accompanied by severe bone disease [51]. These results suggest a significant and interconnected role for DBP-bound 25(OH)D_3_, megalin, and possibly RAP.

Additional knockout mouse models have further highlighted the importance of megalin as an endocytic receptor for vitamin D. The absence of megalin was associated with increased proteinuria, with increased urinary levels of albumin, major urinary protein 6, α_1_-microglobulin, RBP, and DBP compared to control mice, which corresponded to increased excretion of vitamin A (retinol) and 25(OH)D_3_ [50,70]. Further, the Ca^2+^-dependent binding mechanism of megalin appears to be essential, as EDTA treatment abolished ligand binding to megalin [70]. This has direct implications for diseases in which calcium homeostasis is disrupted, such as chronic kidney disease (CKD) and secondary hyperparathyroidism (SHPT), which will be discussed below.

More specific insight towards the contribution of renal megalin was provided through a kidney-specific conditional knockout model. Consistent with global megalin-knockout models, these mice showed enhanced urinary loss of RBP and DBP, and a sixfold reduction in the uptake of 25(OH)D_3_-DBP complexes. This coincided with a 50% reduction in plasma 25(OH)D_3_ and 1,25(OH)_2_D_3_ [71]. Due to reduced vitamin D levels, these mice developed hypocalcemia, dysregulated bone mineralization and severe bone abnormalities [71]. Interestingly, on a vitamin D-normal diet, the kidney-specific megalin knockout mice showed an increase in CYP27B1 and a twofold decrease in CYP24A1 mRNA levels. When placed on a low vitamin D diet, these numbers changed to a 10-fold increase and decrease, respectively [71]. These findings not only suggest that megalin is essential for the retrieval of glomerular filtered vitamin D-DBP complexes, but that compensatory changes in vitamin D-metabolizing enzymes occur to increase circulating and/or tissue levels of 1,25(OH)_2_D_3_ in the absence of functional vitamin D uptake.

**Figure 3:**
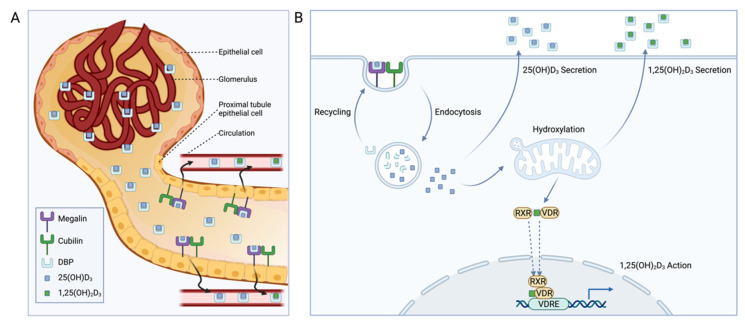
Role of megalin and cubilin in renal vitamin D homeostasis. (**A**) Proximal tubular epithelial cells take up vitamin D-binding protein (DBP)-bound vitamin D metabolites from the glomerular ultrafiltrate through a megalin and cubilin-mediated mechanism [75]. (**B**) After endocytosis within megalin-expressing cells, DBP is degraded and recycled, and 25-hydroxyvitamin D_3_ (25(OH)D_3_) can undergo 1α-hydroxylation to the active 1,25(OH)_2_D_3_ for subsequent vitamin D receptor (VDR) agonism through interactions with the retinoid X receptor (RXR) and VDR response elements (VDREs). Alternatively, endocytosed 25(OH)D_3_ or synthesized 1,25(OH)_2_D_3_ can be secreted to maintain circulating vitamin D homeostasis.

Taken together, these results indicate that renal megalin primarily serves two purposes: (1) preventing urinary loss of vitamin D-DBP complexes and (2) supplying kidney cells with precursor 25(OH)D_3_ for production of the active 1,25(OH)_2_D_3_ (Figure 3). However, some evidence suggests that renal megalin may serve a third purpose related to vitamin D-related gene regulation [72]. Megalin binding protein (MegBP) is an intracellular protein that interacts with the megalin cytoplasmic tail and SKI-interacting protein (SKIP), a component of the VDR transcriptional regulatory complex [72]. As such, May et al. speculate that megalin-mediated endocytosis of vitamin D metabolites may modulate VDR-dependent gene transcription through MegBP and SKIP [73]. Megalin also participates in cell-signaling through regulated intramembrane proteolysis (RIP) [74]. As a result of ligand binding to megalin, the megalin ectodomain is shed, generating a megalin C-terminal fragment (MCTF) that activates γ-secretase, releasing a free C-terminal intracellular megalin domain into the cytosol that is targeted to the nucleus [74]. As this RIP process can be activated by DBP binding to megalin, it is hypothesized that this pathway regulates genes involved in vitamin D metabolism [74]. A more nuanced investigation detailing the physiological relevance of this mechanism or the specific genes affected has yet to be completed.

While the transcriptional impact of direct megalin-mediated intracellular signaling is somewhat unclear, it is well-established that megalin-mediated uptake of vitamin D can modulate vitamin D-metabolizing enzymes, as previously mentioned in DBP-null mice and kidney-specific megalin-null mice [67,71]. These results have been recapitulated in a human cell-derived micro-physiological system, whereby co-administration of 25(OH)D_3_ and 1,25(OH)_2_D_3_ resulted in a dose-dependent increase in 24,25(OH)_2_D_3_ levels, which was significantly impaired by RAP, suggesting that megalin-mediated uptake induces CYP24A1-mediated 24-hydroxylation [76]. Further, megalin may have a minor effect on the semi-selectivity of side-chain 19-nor analogs of 1,25(OH)_2_D_3_, as various forms of vitamin D differentially induced CYP24A1 expression in the absence of renal megalin [77].

The majority of studies regarding the role of megalin in vitamin D metabolism have either focused on global disruptions in megalin, which is problematic due to severe developmental defects allowing only 1–2% of receptor-deficient animals to be viable, or focused solely on renal megalin [51,78]. The contribution of extrarenal megalin towards vitamin D metabolism is discussed below, but it has received less attention, despite lines of evidence suggesting it may be as essential as renal megalin in the context of local, tissue-specific vitamin D action.

## 4. Extrarenal Megalin and Vitamin D Metabolism

The literature surrounding the role of extrarenal megalin in local vitamin D metabolism is limited. However, the studies that have been conducted demonstrate that megalin-mediated endocytosis is essential to functions unrelated to vitamin D in various epithelia, including the testes, vagina, thyroid gland, epididymis, uterus, oviduct, and gallbladder [44,79,80,81,82,83]. As the literature unfolds regarding the multiple functions of extrarenal megalin, many questions arise regarding the specific physiological contribution of these mechanisms and where vitamin D metabolism ranks among them. Functional studies underscore intriguing possibilities for megalin facilitating autocrine and paracrine actions of vitamin D at target tissues. We review all studies to date assessing the functional role of megalin in extrarenal vitamin D metabolism. The existing evidence is suggestive, although somewhat limited to date, in highlighting a clear need for megalin studies focusing on extrarenal target tissues of vitamin D, such as the parathyroid gland, and more physiologically relevant animal models.

### 4.1. Parathyroid Gland

Megalin is expressed in the parathyroid gland and demonstrates functionally important Ca^2+^-binding ability [84,85,86,87]. As megalin is specifically expressed on the surface of parathyroid hormone (PTH)-secreting cells of the parathyroid gland, it may serve as a potential calcium sensor in addition to the calcium-sensing receptor (CaSR) [88]. Whether a possible interaction exists between CaSR and megalin-mediated signaling has not been established, despite the fact that they share Ca^2+^ and vitamin D-related signaling properties [89,90]. A functional calcium-sensing role of megalin in the parathyroid gland may have been uncovered through the mouse monoclonal anti-parathyroid antibody G11, which targets megalin, since exposure to G11 led to the insensitivity of parathyroid cells to extracellular changes in Ca^2+^ for PTH release [91,92,93]. A phosphorylated form of megalin can be immunoprecipitated from cultures of primary human parathyroid cells, underscoring an additional, albeit speculative, role of megalin in phosphate sensing [29,84]. The expression of parathyroid megalin mRNA and protein is reduced in pathological parathyroid adenomas in patients with primary hyperparathyroidism, and is associated with aberrant Ca^2+^ regulation, highlighting the immediate relevance of parathyroid megalin in pathologies, such as CKD and SHPT [42,94]. The role of parathyroid gland megalin in vitamin D metabolism has not yet been investigated. The parathyroid gland is significantly involved in the pathogenesis of CKD and SHPT, as well as a target of vitamin D receptor agonist (VDRA) therapy; thus, it is important that further studies focused on parathyroid megalin be undertaken.

### 4.2. Mammary Gland, Prostate, and Colon

Mammary epithelial cells express vitamin D-metabolizing enzymes such as CYP27B1 and CYP24A1, and have been shown to metabolize 25(OH)D_3_ to 1,25(OH)_2_D_3_ [22,95]. Rowling et al. sought to elucidate how 25(OH)D_3_-DBP complexes gain access to mammary cells. Using temperature-shift techniques, they observed the uptake of fluorophore-conjugated DBP into mammary cells at 37 °C, a temperature conducive to endocytosis, which was abolished at 4 °C, a microtubule-disrupting temperature. The essential role of megalin was confirmed using RAP, which significantly blunted DBP uptake into mammary cells at 37 °C. These data suggest that the internalization of DBP into mammary cells may occur via megalin-mediated endocytosis. Furthermore, endocytosed 25(OH)D_3_-DBP activated a CYP24A1 reporter gene, demonstrating that megalin-mediated endocytosis of vitamin D-DBP induces vitamin D-metabolizing enzyme activity in extrarenal tissues [96].

These findings have been recapitulated by Chlon et al., who showed physiologically-relevant megalin-mediated endocytosis in mammary cells, and modulation of megalin expression and action by retinoids [97]. Treatment of mammary cells with 10 μmol/L all-trans-retinoic acid (RA), or RA and 100 nmol/L 1,25(OH)_2_D_3_ in combination resulted in a 1.8-fold and 4.2-fold increase in megalin mRNA levels, respectively. Interestingly, RA did not significantly increase cubilin mRNA but elevated Dab2 mRNA, an essential intracellular protein for renal megalin function, by 6.2-fold [98]. The induction of megalin and Dab2 mRNA was consistent with significantly enhanced uptake of DBP in RA-supplemented media, supporting a meaningful role for both megalin-mediated uptake in the mammary gland alongside retinoid stimulation of megalin mRNA and function. This data also underscores a relationship between vitamin D and megalin, as 1,25(OH)_2_D_3_ alone enhanced megalin mRNA, which may correspond to stimulated uptake of vitamin D into target tissues [97]. Similar findings have been observed in prostate and colon epithelial cells. Following treatment with 10 μmol/L RA, megalin and Dab2 mRNA expression increased by approximately 3-fold in the tested prostate and colon cell lines [99]. RA-mediated increases in megalin and Dab2 expression coincided with enhanced megalin-mediated uptake, which again was inhibited at 4 °C. Immunofluorescence assays demonstrated punctate colocalization of DBP and megalin around the perimeter of prostate cells, suggesting the subcellular localization of megalin is physiologically relevant to its function. These results provide evidence that prostate and colon megalin are functional in internalizing vitamin D-DBP complexes and that its activity is inducible by RA [99].

### 4.3. Muscle and Fat

Megalin and cubilin expression were identified in differentiated muscle cells but not in undifferentiated myoblasts [100]. Interestingly, the time-dependent uptake of isotopically-labelled 25(OH)D_3_ was 2- to 3-fold higher in differentiated myotubes than in myoblasts, suggesting that megalin promotes the uptake of vitamin D into differentiated muscle cells, and that differentiation itself may moderate megalin function [100]. The function of megalin in myotubes was confirmed via RAP inhibition of megalin, which reduced the uptake of 25(OH)D_3_ into myotubes by 66% over a 16-h incubation [100]. These results were recapitulated by the same group, who again observed a significantly higher uptake of 25(OH)D_3_ in myotubes than undifferentiated myoblasts at 4 or 16 h, consistent with their high and low megalin expression, respectively [101]. In their analysis of fat cells, pre-adipocytes demonstrated significant uptake of 25(OH)D_3_ at 4 and 16 h, and differentiated adipocytes did not, which paralleled their strong and negligible megalin expression, respectively [101]. As skeletal muscle consists primarily of differentiated muscle cells and adipose tissue primarily of differentiated fat cells, these results suggest that megalin-mediated endocytosis of vitamin D-DBP complexes is a predominant mechanism of vitamin D uptake in muscle, but not in fat tissue.

### 4.4. Mesenchymal Stem Cells

As both muscle and fat cells are derived from mesenchymal stem cells, it is of interest to uncover the role of megalin within them. Gao et al. demonstrated that megalin is essential for the biosynthesis of 1,25(OH)_2_D_3_ and stimulation of VDR and osteoblastogenesis target genes in human mesenchymal stem cells (hMSCs) [102]. In comparing hMSCs with high or low constitutive megalin expression, the biosynthesis of 1,25(OH)_2_D_3_ in hMSCs with low megalin was shown to be 46% of hMSCs with high megalin [102]. This corresponded to osteoblastogenesis induction, whereby incubating hMSCs with 25(OH)D_3_ induced expression of osteoblast signature genes Runx2 and ALP in hMSCs with high megalin, but not with low megalin, suggesting that megalin is required for both intracellular synthesis of 1,25(OH)_2_D_3_ from substrate 25(OH)D_3_, and 25(OH)D_3_-mediated induction of osteoblastogenesis [102]. These results were further supported by siRNA-mediated knockdown of megalin, whereby 1,25(OH)_2_D_3_ synthesis from 25(OH)D_3_ was reduced by 77% in siRNA-exposed hMSCs [102]. Correspondingly, 25(OH)D_3_ stimulated Runx2, ALP, and BSP expression in control hMSCs, but not in hMSCs exposed to megalin siRNA [102]. Similar results were observed for CYP24A1 after exposure to 25(OH)D_3_, suggesting that megalin is required to induce VDR target genes by the 25(OH)D_3_-DBP complex. It is of interest that exposure of hMSCs to 1,25(OH)_2_D_3_ in either the low megalin or siRNA-mediated knockdown experiments did not lead to differential effects on osteoblastogenesis or induction of VDR target genes, suggesting that the actions of 1,25(OH)_2_D_3_ in hMSCs are independent of megalin-mediated endocytosis. The differences in megalin handling of 25(OH)D_3_ and 1,25(OH)_2_D_3_ were also observed in mammary epithelial cells [96]. Thus, the expression of megalin in hMSCs and mammary epithelial cells appears necessary for the uptake and conversion of 25(OH)D_3_ to 1,25(OH)_2_D_3_.

### 4.5. Bone

Primary human osteoblast cells have been found to express CYP27B1 mRNA and secrete detectable levels of 1,25(OH)_2_D_3_ in response to 25(OH)D_3_ exposure [103]. Exposure of these cells to physiological levels of 25(OH)D_3_ (20–100 nM) coincided with stimulation of CYP24A1 mRNA and VDR target genes related to osteogenesis, such as osteocalcin, osteopontin and RANKL [103]. Further, siRNA-mediated knockdown of CYP27B1 almost wholly abolished the response of osteoblasts to 25(OH)D_3_, supporting local 25(OH)D_3_ conversion and activation to 1,25(OH)_2_D_3_. While this study did not assess the functional role of megalin in facilitating these actions, megalin mRNA was detected in the human osteoblast cells [103]. These results not only parallel those discussed earlier in terms of the stimulatory capacity of 25(OH)D_3_ in extrarenal tissues but also suggest that megalin-mediated endocytosis of 25(OH)D_3_ promotes the autocrine synthesis of 1,25(OH)_2_D_3_ at target tissues, which has functional regulatory consequences for these tissues. A receptor-mediated mechanism for DBP uptake has also been observed for human B-lymphoid cells; however, whether megalin is the receptor involved remains unclear [104].

## 5. Megalin in Chronic Kidney Disease

Many diseases have been associated with maladaptive alterations in megalin expression or function, or the opposite, where megalin dysfunction leads to various pathological states, summarized by Nielsen et al. [27]. The scope of this section will henceforth be focused on the vitamin D-related implications of altered megalin expression in kidney diseases.

### 5.1. Chronic Kidney Disease Overview

CKD is a disease of great interest, given its enormous costs to patient well-being and health care systems; consequently, its intimate relationship with abnormal vitamin D metabolism has been extensively explored. CKD is clinically defined by a chronic reduction in kidney function and is associated with the pathogenesis of SHPT, the pathological rise in PTH. CKD is associated with decreased levels of the active form of vitamin D, 1,25(OH)_2_D_3_, leading to reduced VDR-mediated suppression of PTH secreted by the parathyroid gland and contributing to SHPT development [105]. Treatment of SHPT commonly involves the administration of VDRAs, such as 1,25(OH)_2_D_3_. However, these treatments can become problematic in patients, either indirectly mediating off-target effects, such as left ventricular hypertrophy or, most notably, the emergence of VDRA resistance [106]. Approximately 20–30% of SHPT patients become resistant to treatment with VDRAs, as serum PTH does not decrease despite increasing doses of VDRAs, representing a state of ‘active vitamin D failure,’ that may exacerbate cardiovascular toxicity [107]. The mechanisms of treatment-acquired vitamin D resistance are incompletely understood, with contributions potentially arising from impaired function or expression of VDR, retinoid X receptor (RXR), increased uremic toxins, elevated parathyroid calreticulin, alterations in vitamin D-metabolizing enzymes (CYP2R1, CYP27A1, CYP27B1) or DBP [108,109]. The contribution of megalin towards vitamin D resistance as a receptor for vitamin D substrate supply for the parathyroid gland has, to date, received little attention in the literature.

### 5.2. Megalin in Chronic Kidney Disease

A partial nephrectomy rat model of CKD demonstrated a gradual decrease of megalin mRNA in the remnant kidney as early as week 2, which continued to decline throughout the study [110]. This was accompanied by an increase and decrease in CYP27B1 and CYP24A1 mRNA, respectively. By week 8, the levels of CYP27B1 mRNA were significantly elevated, VDR mRNA was reduced, and the ratio of CYP27B1 to CYP24A1 and megalin mRNA was more pronounced [110]. As indicated above, the up and downregulation of vitamin D activating and deactivating enzymes suggest a compensatory mechanism to enhance 1,25(OH)_2_D_3_ biosynthesis and preservation in the absence of megalin-mediated endocytosis. Interestingly, the plasma levels of calcium, phosphate, and 1,25(OH)_2_D_3_ were not significantly different from controls in the study duration, while PTH was elevated considerably [110]. Thus, it is unclear whether this was due to compensatory up and downregulation of CYP27B1 and CYP24A1, a non-megalin-mediated mechanism of 1,25(OH)_2_D_3_ synthesis, or an early state of SHPT attempting to compensate for impaired mineral and vitamin D handling [111,112]. In a transgenic mouse model overexpressing renin to recapitulate CKD, megalin protein expression in proximal tubules was significantly decreased but returned to normal levels following angiotensin II type 1 (AT1) receptor antagonism [113]. These results were concordant with angiotensin II-mediated suppression of megalin mRNA and protein in another CKD model, followed by impaired megalin-mediated uptake [114]. As angiotensin II levels are elevated in CKD, this data suggests a potential mechanism by which megalin expression and function are inhibited in kidney diseases, potentially leading to detrimental effects on vitamin D metabolism [115]. In a 5/6 nephrectomy rat model of CKD, megalin expression was significantly reduced in the renal cortex after 8 weeks, but the expression of cubilin remained unchanged [116]. The physiological relevance of a differential renal expression of megalin and cubilin is unclear, however, as uptake functionality was not assessed. Human kidney biopsies from patients with CKD and Fabry nephropathy also show reduced megalin and cubilin expression compared to healthy controls [117]. However, reports of decreased renal megalin expression in CKD are not consistent. In another nephrectomy rat model of CKD, the megalin and cubilin protein levels were increased relative to controls [118]. As these findings were based on a qualitative assessment of an immunohistochemical characterization of kidney tissue, they should be interpreted cautiously. Nonetheless, they suggest that further studies must be conducted to elucidate the pathophysiologic role of decreased megalin expression in a CKD setting.

A recent study suggests that megalin dysfunction may, in itself, contribute to CKD progression; in a kidney-specific knockout model of megalin, the glomerular filtration rate (GFR) decreased, and plasma creatinine increased, both being clinical indicators of CKD [119,120]. Similar findings were observed in a clinical cohort of patients with pathogenic megalin mutations, where, in addition to classical signs of renal decline, enhanced proteinuria of DBP and other megalin ligands, and glomerular and tubulointerstitial pathohistological lesions, were observed [119,121].

### 5.3. Vitamin D Regulation

**Figure 4:**
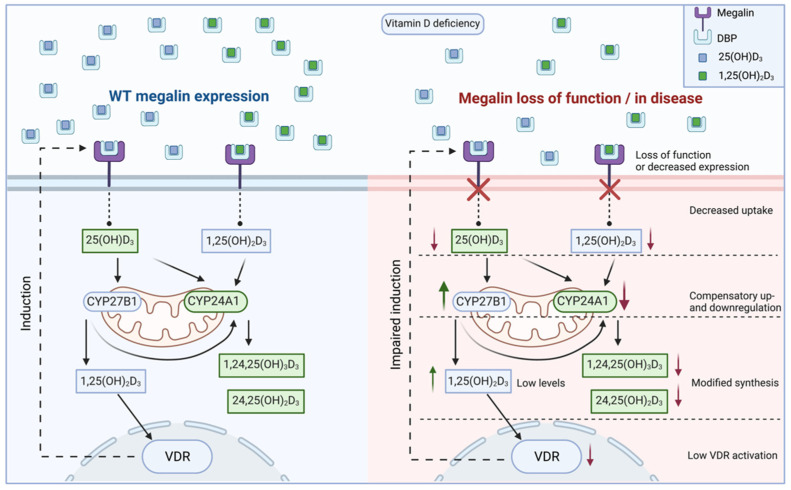
Involvement of megalin in vitamin D metabolism and proposed effect of kidney disease or loss of function. After megalin-mediated endocytosis, 25-hydroxyvitamin D_3_ (25(OH)D_3_) may be sent to the mitochondria for cytochrome P450 (CYP)-mediated inactivation or CYP27B1-mediated activation. 1,25-dihydroxyvitamin D_3_ (1,25(OH)_2_D_3_), either endocytosed or intracellularly generated, may be inactivated via CYP24A1, or interact with the vitamin D-receptor (VDR) to exert intracrine genomic effects such as upregulating megalin gene expression. Alternatively, vitamin D metabolites may re-enter the circulation to exert endocrine effects. Evidence from knockout models and models of kidney disease suggests megalin function or expression is decreased, leading to a reduced capacity for endocytosis. In a setting of vitamin D deficiency, this leads to the compensatory up and downregulation of CYP27B1 and CYP24A1, respectively, modifying the synthesis of vitamin D metabolites. Low levels of 1,25(OH)_2_D_3_, due to decreased uptake, may cause lower VDR activation and reduced megalin expression. For visual clarity, only the VDR genomic effect on megalin expression has been displayed, but it is conceivable that many other expression profiles would be altered due to impaired megalin endocytosis.

As CKD seems to induce alterations in megalin expression, vitamin D-mediated regulation of megalin becomes relevant, given the relationship between CKD and reduced 1,25(OH)_2_D_3_ levels. Megalin mRNA and protein were stimulated after exposing rat PTECs to 1,25(OH)_2_D_3_ and RA [97,122], suggesting a “vicious cycle” hypothesis in the context of CKD. Assuming 1,25(OH)_2_D_3_ can stimulate renal megalin expression, this would ensure maintenance of systemic vitamin D levels via megalin-mediated endocytosis. In CKD, where levels of 1,25(OH)_2_D_3_ levels are reduced, this would lead to decreased megalin expression, reducing the capacity of renal 25(OH)D_3_ uptake and further reducing 1,25(OH)_2_D_3_ production, propagating the cycle and exacerbating vitamin D deficiency (Figure 4) [123]. This may, partially, explain the phenotype of abnormalities in CKD-mineral and bone disorder (CKD-MBD), in which reduced levels of 1,25(OH)_2_D_3_ promotes hypocalcemia, stimulating excessive PTH production and, in turn, accelerated bone turnover [124]. Other reviews by Dusso, Kim and Kim, and Bosworth and de Boer have discussed this concept [125,126,127,128]. This hypothesis has been challenged by a more recent study using a human cell-derived micro-physiological system, in which exposure to 1,25(OH)_2_D_3_ did not induce upregulation of megalin but instead demonstrated a significant downward trend of megalin mRNA expression [76]. While these results are interesting, statistically significant suppression of megalin mRNA was only achieved at a supraphysiological dose of 1,25(OH)_2_D_3_ at 500 nM. This dose is well above the upper limit of circulating levels and at a level consistent with 1,25(OH)_2_D_3_ toxicity, where pro-apoptotic effects, angiogenesis, invasion, and metastasis are observed [129,130,131,132]. Precluding the possibility that it was high-dose exposure of 1,25(OH)_2_D_3_ mediating toxic effects on megalin mRNA, these results highlight potential interspecies variability involving megalin regulation within humans and rodents and challenge the relationship of 1,25(OH)_2_D_3_ regulation of megalin expression. The concept of interspecies variability in vitamin D handling is not foreign, as differences between humans and rodent models have also been demonstrated with urinary DBP excretion. For example, urinary DBP excretion was not found to be increased in cubilin-deficient mice; however, it was raised in both megalin-deficient and cubilin-deficient patients [60,61,121]. Although limited, these data suggest that reabsorption of DBP in mice can occur without cubilin, whereas it may play a more significant role in humans.

### 5.4. Diabetic Nephropathy

A major cause of CKD in patients is diabetic nephropathy, which develops in approximately 40% of patients with diabetes [133]. Diabetic nephropathy induces a similar inhibition of megalin and Dab2 mRNA to CKD, with approximately 50 and 80% reductions in mRNA, respectively, whereas cubilin mRNA remains unchanged [134]. Reductions in megalin and Dab2 mRNA correspond to increased DBP, 25(OH)D_3_, and 1,25(OH)_2_D_3_ urinary excretion in diabetic fatty rats, concomitant with reduced serum levels of both 25(OH)D_3_ and 1,25(OH)_2_D_3_ [134]. Dysregulation of intrarenal vitamin D metabolism has been similarly reported in a mouse model of diabetic nephropathy, consistent with increased excretion of DBP, 25(OH)D_3_, megalin itself, and elevated CYP27B1 mRNA [135]. Other animal studies have also reported decreased proximal tubule megalin protein expression in rat models of diabetic nephropathy [136,137]. In patients with type 1 diabetes, a significant elevation in the urinary excretion of low molecular weight proteins, DBP and megalin was observed that paralleled the magnitude of vitamin D deficiency in these patients, linking megalin dysfunction to impaired vitamin D homeostasis [138,139]. The findings of increased urinary megalin excretion raise meaningful implications for kidney disease, as full-length urinary megalin (C-megalin) excretion is related to the pathogenesis of diabetic nephropathy [140,141]. The significance of urinary C-megalin excretion in vitamin D metabolism was assessed in pre-dialysis CKD patients, where it was negatively associated with serum levels of 25(OH)D_3_, 1,25(OH)_2_D_3_, and 24,25(OH)_2_D_3_ [142]. These results suggest that impaired megalin-mediated endocytosis of 25(OH)D_3_-DBP complexes contributes to dysregulated systemic vitamin D homeostasis. Both diabetic nephropathy and CKD likely impair the reabsorption of 25(OH)D_3_-DBP complexes via decreased megalin protein and mRNA expression, or increased urinary excretion, ultimately comprising hypovitaminosis D.

## 6. Summary and Outlook

The role of the endocytic receptor megalin in reabsorbing DBP-bound vitamin D metabolites from the renal proximal tubule has been well established, confirming its significance in the metabolism and homeostasis of circulating vitamin D. It is well known that vitamin D-metabolizing machinery is widely expressed in the body and can take up 25(OH)D_3_ for local conversion into 1,25(OH)_2_D_3_ via CYP27B1. However, little attention has been given to the mechanisms of extrarenal 25(OH)D_3_ uptake and how these mechanisms are modified in diseases. In addition to the kidney, megalin is widely expressed in several cell types, including mammary cells, osteoblasts, muscle, fat, mesenchymal stem cells, thyroid, and parathyroid cells, among many others. Not only does this expression coincide with the expression of vitamin D-metabolizing enzymes, but functional studies have demonstrated through knockout models, or RAP-mediated inhibition, that megalin-mediated endocytosis is essential for the uptake of vitamin D into cells, and its impairment leads to maladaptive alterations in vitamin D metabolism. However, the understanding of extrarenal megalin is quite incomplete, with essential calciotropic tissues expressing megalin, such as the parathyroid gland, not having been investigated sufficiently, thereby leaving gaps in knowledge regarding the contribution of megalin to tissue-specific 1,25(OH)_2_D_3_ production. More studies about the role of megalin in these tissues are needed, as megalin dysfunction has been associated with vitamin D deficiency, and evidence suggests that megalin is critical to the progression of dysregulated vitamin D metabolism in CKD and other kidney diseases. The specific contribution of megalin in the pathogenesis of CKD remains elusive, however, as not all findings of megalin expression and regulation by 1,25(OH)_2_D_3_ are consistent. As analogs of vitamin D are given as treatments for conditions like SHPT in CKD, the complications arising from the free hormone hypothesis, inconsistent 1,25(OH)_2_D_3_ regulation of megalin and the incompletely understood mechanisms of megalin regulation in disease and extrarenal tissues all suggest the need for additional research assessing the role of megalin in vitamin D homeostasis. Megalin is very likely an important player in local networks of vitamin D metabolism within extrarenal tissues.

## Abbreviations

1,25(OH)_2_D_3_	1,25 dihydroxyvitamin D_3_
25(OH)D_3_	25 hydroxyvitamin D_3_
24,25(OH)_2_D_3_	24,25 dihydroxyvitamin D_3_
1,24,25(OH)_3_D_3_	1,24,25 trihydroxyvitamin D_3_
ALP	Alkaline phosphatase
AMN	Amnionless
AP-2	Adaptor protein-2
ARH	Autosomal recessive hypercholesterolemia
AT1	Angiotensin II type 1
CaSR	Calcium-sensing receptor
CKD	Chronic kidney disease
CYP24A1	Cytochrome P450 24A1
CYP27B1	Cytochrome P450 27B1
Dab-2	Disabled-2
DBP	Vitamin D-binding protein
EGF	Epidermal growth factor
GIPC	GAIP interacting protein
GAIP	G alpha interacting protein
hMSC	Human mesenchymal stem cell
MegBP	Megalin binding protein
PTEC	Proximal tubular epithelial cells
PTH	Parathyroid hormone
RA	All-*trans*-retinoic acid
RAP	Receptor-associated protein
RBP	Retinol-binding protein
RIP	Regulated intramembrane proteolysis
RXR	Retinoid X receptor
SHPT	Secondary hyperparathyroidism
SKIP	SKI-interacting protein
VDR	Vitamin D receptor
VDRA	Vitamin D receptor agonist
VDRE	VDR response element
